# Like Mother(-in-Law) Like Daughter? Influence of the Older Generation’s Fertility Behaviours on Women’s Desired Family Size in Bihar, India

**DOI:** 10.1007/s10680-016-9379-z

**Published:** 2016-04-06

**Authors:** Abhishek Kumar, Valeria Bordone, Raya Muttarak

**Affiliations:** 1 International Institute for Population Sciences (IIPS), Mumbai, India; 2 Centre for Research on Ageing, University of Southampton, Southampton, UK; 3 Wittgenstein Centre for Demography and Global Human Capital (IIASA, VID/ÖAW, WU), International Institute for Applied Systems Analysis (IIASA), Schlossplatz 1, 2361 Laxenburg, Austria

**Keywords:** Coombs scale, Desired family size, Fertility preference, India, Intergenerational transmission, Mother-in-law

## Abstract

This paper investigates the associations between preferred family size of women in rural Bihar, India and the fertility behaviours of their mother and mother-in-law. Scheduled interviews of 440 pairs of married women aged 16–34 years and their mothers-in-law were conducted in 2011. Preferred family size is first measured by Coombs scale, allowing us to capture latent desired number of children and then categorized into three categories (low, medium and high). Women’s preferred family size is estimated using ordered logistic regression. We find that the family size preferences are not associated with mother’s fertility but with mother’s education. Mother-in-law’s desired number of grandchildren is positively associated with women’s preferred family size. However, when the woman has higher education than her mother-in-law, her preferred family size gets smaller, suggesting that education provides women with greater autonomy in their decision-making on childbearing.

## Introduction

It is well documented that parents play an important role in shaping the preferences and behaviours of young adults. A wide array of studies, mainly in Western developed countries, has shown such intergenerational transmission with respect to fertility preferences (Axinn et al. 1994; Bühler and Philipov 2005) and number of children ever born (Murphy and Knudsen 2002; Murphy and Wang 2001). Continuities in parent–child fertility have implications for population size and structure since people born into large families are themselves more likely to make an above-average contribution to successive generations. According to the principle of linked lives (Elder 1977, 1994), parental behaviours during the children’s life course significantly influences both desires and behaviours of children in adulthood. Empirical evidence has confirmed that individuals learn and incorporate the preferences and behaviours of relevant others in order to make their own decisions. Likewise, research on less-developed countries has shown that living in an extended family is associated with higher fertility (Easterlin 1978), but so far very little is known about the intergenerational transmission of fertility preferences in such contexts. Studies on intergenerational transmission of fertility in non-Western countries are rare (Murphy 2013). Recent evidence, however, shows a positive though small intergenerational correlation between women’s completed fertility and that of their parents across 46 sub-Saharan Africa, Asian and Latin American countries (Murphy 2012).

The current paper contributes to this line of research by studying the influence of mother’s and mother-in-law’s fertility behaviour on young women’s fertility preferences in rural Bihar, a north-eastern state of India, using primary data collected in 2011. According to the socialization of value’s perspective (Preston 1976), ideals and preferences on childbearing in the parent generation are important determinants of children’s fertility. However, in Indian society, especially in the north where the practice of patrilineal descent and patrilocal residence is widespread, young married women typically move to live with their husband’s family and are absorbed into their husband’s lineage (Jejeebhoy and Sathar 2001). In such a context, men hold a central position in the family while older women, i.e. mother-in-law of the young bride, wield the main authority over household affairs (Das Gupta et al. 2003). This leaves the young bride little autonomy over her family life which in turn translates into the intergenerational transmission of fertility that is likely different from that of Western societies. This study hence aims to shed light on intergenerational transmission mechanisms in rural areas of north-eastern India, where fertility remains well above the replacement rate, contraception use is not widespread, and young women are rather confined in the secluded environment of their husband’s family.

Note that this study focuses on women’s family size preferences, i.e. small versus large family size. The concept of preferences here refers to what people want or would like, and it may therefore be thought of as being unconstrained. We acknowledge that, given women’s current status, they may incorporate individual’s personal circumstances and perceptions in forming their preferences (Miller and Pasta 1995; Thomson 1997). Understanding women’s family size preferences is nevertheless important since they are the basic link in the chain leading to reproductive decision-making (Philipov and Bernardi 2012) and behaviour (Schoen et al. 1999).

The remainder of the paper is organized as follows. We first outline our hypotheses derived from the theoretical and empirical literature. The next section describes the data and the study sample. After explaining our empirical strategy, we present descriptive and multivariate results. In the last section, we conclude with a discussion of our findings.

## Theoretical Framework and Hypotheses

The concept of “linked lives” (Elder 1977, 1994) from the life course perspective is useful in summarizing how the experiences of one generation affect the experiences of successive generations. In particular, the literature on intergenerational transmission of fertility has mainly focused on the family of origin effect in terms of sib-ship size; that is, women with more siblings are expected to have more children than their counterparts with a lower number of brothers and sisters (Murphy 1999; Murphy and Knudsen 2002). While the role of genetics in the transmission of both the desire for children and the ability to have children was emphasized in early works (Imaizumi et al. 1970), later studies also showed how the intergenerational transmission of socioeconomic conditions such as education, income, standard of living and pattern of consumption shapes opportunities as well as constraints related to childbearing behaviours (Bengtson 1975; Ben-Porath 1975). Parents can therefore influence fertility preferences and behaviour of children via both direct and indirect channels throughout the life course.

With respect to preferences for family size, socialization and social pressure are two main mechanisms explaining transmission across generations. Children are likely to learn through observing the behaviours and experiences of the parents (Kohler et al. 2001; Montgomery and Casterline 1996) and to form their preferences according to the values and norms related to marriage and family into which they are socialized (Anderton et al. 1987; Keim et al. 2009; Kolk 2014; Preston 1976). According to the theory of planned behaviour, people also experience social pressure from their social environment to engage in certain rather than other behaviours, and this leads to the formation of subjective norms (Ajzen 1991). Fertility norms thus are enforced through group pressure and culture.

While intergenerational transmission of social status is often driven by social origin of fathers (Graaf and Kalmijn 2001), mothers appear to have a more important role in transferring fertility-related characteristics such as timing of childbearing, family size preference and number of children ever born (Axinn et al. 1994; Barber 2001; Barber and Axinn 1998). Typically, in Western societies mothers serve as significant others from whom daughters learn and observe. Moreover, mothers provide immediate care, advise on reproductive issues and social support to young women after marriage (Chan and Elder 2000; Dubas 2001; Pollet et al. 2009). Following the previous evidence, we therefore expect a positive association between the number of children ever born to the mother and the woman’s fertility preferences. We derive the first hypothesis as follows:

### **H1**

Fertility of biological mother is positively associated with women’s family size preferences.

Social interaction with others outside the family of origin may also be a crucial source of transmission and diffusion of innovations such as modern contraceptives (Bernardi 2003; Kohler 2001). Indeed, empirical evidence from developed societies has shown a positive correlation between fertility behaviours among siblings (Kuziemko 2006; Lyngstad and Prskawetz 2010), co-workers (Asphjell et al. 2013; Ciliberto et al. 2013; Pink et al. 2014) and peers (Balbo and Barban 2014; Manski and Mayshar 2003), pointing to both the direct and indirect influence of social networks on women’s fertility. However, the composition of social network members may vary according to sociocultural contexts. While going to college or participation in the labour force enables a woman to build up her networks beyond close kin, in cultural settings such as those in north-eastern India considered in this study, female mobility is often limited (Self and Grabowski 2013). Subsequently, women’s social interactions are constrained within the domestic domain.

Unlike the south of India, where endogamous marriage within the community and extended family is commonly practised, Bihar and the north-eastern states in general are characterized by exogamous marriage in which the new bride is isolated from her kinship network and becomes dependent on her in-laws (Dyson and Moore 1983). Accordingly, a newly married young woman who moves into the in-laws house becomes “re-socialized” so that she will identify her own interests with those of her husband and, in particular, of his mother. Indeed, it is in close proximity with the mother-in-law that she will spend most of the day (Jejeebhoy and Sathar 2001).

It is highly possible that, in the context under study, a young wife’s fertility preference is shaped by what she observes in her husband’s family given the relatively young age at marriage for women—often lower than the legal age of 18. The mean age at marriage for girls in Bihar is 17.6 years compared to 19.8 for the country average. Correspondingly, 68.2 % of currently married women aged 20–24 were married before age 18 in Bihar compared to 42.9 % in India based on the 2007–2008 District Level Household and Facility Survey (IIPS 2010a, 2010b). It is thus highly possible that the fertility behaviour of the mother-in-law may influence women’s family size preference owing to the young age at marriage coupled with the fact that a young bride usually moves in with the husband’s family (Martin et al. 1999). We thus derive the following hypothesis:

### **H2**

In the rural Indian context under focus in this analysis, fertility of mother-in-law is expected to have greater influence on women’s family size preferences than mother’s fertility.

Individuals not only learn from their significant others, but they also try to respect the social norms shared within their social environment in order to get approval and avoid conflict with their kin and peers. These norms are defined as “what is typical or normal, thus, what most people do” (Cialdini et al. 1990). Subsequently, this becomes what is most “sensible to do”. Individuals whose significant others have many children may thus be more prone to prefer a large family. Although social norms constructed and perpetuated in large social groups can be considered as an independent mechanism of social influence, the small scale of the villages we investigate allows us to consider social norms as part of the social pressure dynamic (for a similar argument, see Balbo and Barban 2014). The strength of such social pressure depends on the homogeneity of the networks. In highly connected, homogenous networks as the one we are looking at, the ability of the woman to decide freely or deviate from prevailing norms is likely to be low.

In the northern Indian context where women have limited control over their lives, the in-law family may serve as a medium for the enforcement of norms related to fertility because it possesses considerable sanctioning power (e.g. disapproval). Close interactions between mothers-in-law and young wives may therefore channel decisions on household matters, including procreation, through a particularly prominent social pressure from the in-laws (Säävälä 2001). Living with the mother-in-law, a young woman in Bihar has little autonomy, limited access to economic resources, lack of freedom of mobility and a limited role in household decision-making compared to cases where she or the spouse is the head of the household (Bloom et al. 2001; Cain et al. 1979). In such context, the influence of mothers-in-law on young couples’ family planning decision is not negligible, as evident in Pakistan and rural areas in India in general (Fikree et al. 2001; Kadir et al. 2003; Wilson-Williams et al. 2008).

Living under the authority of the mother-in-law can also be a barrier to a woman’s knowledge of and access to modern contraceptives. Indeed, large family size norm and son preference coupled with lack of knowledge of modern contraceptive methods are the main reasons for low use of contraception in Bihar, the region with the highest fertility and highest unmet contraceptive need in India (Bhatnagar et al. 2013; IIPS 2010a; RGI 2011). Contraceptive prevalence in Bihar among women aged 15–24 years remains negligible (only 12.6 % using any method and 4.0 % using non-terminal modern method according to the 2005–2006 National Family Health Survey) with as high as 32 % unmet need for contraception (Santhya and Jejeebhoy 2012). Contraceptive use among the older age groups appears to be higher, but it is mainly because once women achieve their desired fertility, they adopt a permanent method (sterilization) (Daniel et al. 2008)

Given that previous studies have documented a dominant role mothers-in-law play in young women’s contraceptive use decisions (Char et al. 2010) and in exerting a strong pressure to bear sons (Bhat and Zavier 2003; Das 1987), we expect the woman’s family size preference to be strongly influenced by that of mother-in-law. The third hypothesis thus is described as follows:

### **H3**

The number of grandchildren that the mother-in-law would like to have from the women interviewed strongly influences the latter’s fertility preferences.

Apart from the influence of significant others on women’s preferred family size, other characteristics such as age, religion, caste and education have been underscored as the key to fertility decline during the so-called demographic transition at both the population (Bongaarts 2003, 2010) and individual level (Borkotoky and Unisa 2014; Kravdal 2002). Since higher educated women tend to marry later and bear higher opportunity costs of childbearing than their lower educated counterparts, they may in turn have lower fertility than the latter. Meanwhile, education is also negatively associated with infant mortality (Pamuk et al. 2011). The increase in the chances of infant surviving consequently reduces the necessity to bear many children.

In countries undergoing development, education also enables women to have control over resources and their own lives (Basu 2002). Indeed, a recent literature review on the association between women’s empowerment and fertility in a less-developed country context confirms a robust relationship between the two factors (Upadhyay et al. 2014). In general, it is found that empowerment (measured as women’s participation in household decision-making, freedom of mobility and educational attainment) is associated with lower fertility, longer birth intervals, lower rates of unintended pregnancy and smaller ideal family size. Like in other societies such as sub-Saharan Africa (Bankole and Singh 1998; Dodoo 1993; Isiugo-Abanihe 1994; Tilahun et al. 2014), it is found also in India (Das et al. 2013; Singh et al. 2006) that men generally desire a higher number of children than their wives. In this regard, women’s empowerment may increase a wife’s negotiation power over childbearing issues, including smaller family size.

Likewise, in the South Asian context, it has been documented that mothers-in-law are often the main barrier to the utilization of modern contraceptive methods (Masood Kadir et al. 2003; Stephenson and Hennink 2004). Accordingly, an increase in female autonomy may allow women to make their fertility choices as they wish. Given that education is one of the key factors contributing to women’s empowerment, the fourth hypothesis is the following:

### **H4**

Social pressure from mother-in-law and husband is lower when the woman has higher education.

## Data and Methods

### The Primary Data on Women and Their Mother-in-Law

**Fig. 1:**
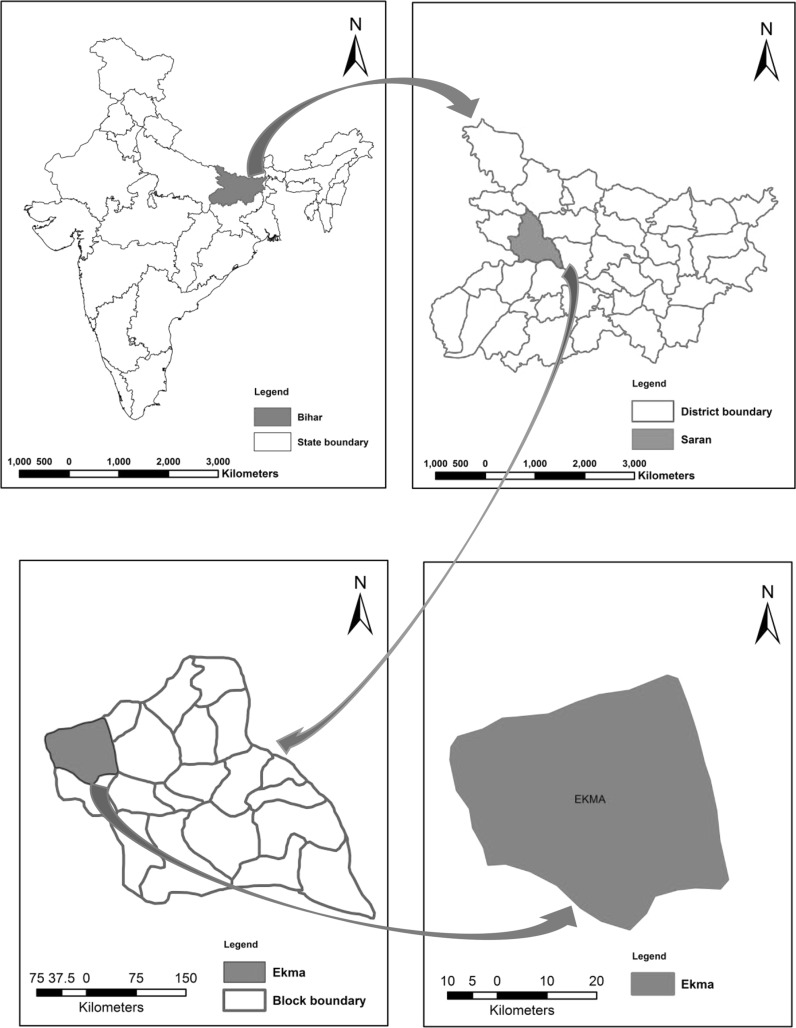
Location map of the study area

The present study is based on primary data from pairs of women and mother-in-law collected in 2011 by one of the authors in a rural area of India. Each woman–mother-in-law pair was co-residing and sharing the same kitchen at the time of interview. The three villages from which the sample was selected, i.e. Bareja, Nautan and Sheetal Pur, as we will explain later, are located in the *Ekma* block (administrative unit in which districts are divided) of the Saran district. The Saran district belongs to the state of Bihar and is located in the north-eastern part of India (Fig. 1).

Within India, Bihar ranks fairly low in terms of socioeconomic indicators and is considered to be the least developed state based on its demographic characteristics. Bihar is the third most populous state of India and has experienced the highest population growth rate in the last decade (Office of the Registrar General and Census Commissioner, India 2011). Bihar’s total fertility rate (TFR = 3.5 in 2012) is one of the highest in India (MoHFW 2013). Meanwhile, contraceptive prevalence rate in Bihar (34 % in 2007–2008) is one of the lowest among the major states (IIPS and MoHFW 2010).

The sample was selected using a three-stage sampling design. In the first stage, the *Ekma* block within the Saran district was chosen since its socioeconomic characteristics are very close to Bihar’s average. We focus on rural villages because the vast majority of the population (90 %) in Bihar resides in rural areas (Office of the Registrar General and Census Commissioner, India 2011). In the second stage, the three villages with the highest number of households were selected in order to ensure socioeconomic heterogeneity in the sample. Villages were considered as Primary Sampling Units (PSUs). In order to obtain the desired sample size (*N* = 450), a complete house-listing of the selected PSUs was drawn up. In the third stage, only those households with the following characteristics were selected as eligible for interview: the interviewed woman (1) was married; (2) had at least one child; and (3) was residing and sharing the same kitchen with the mother-in-law at the time of interview. The choice to focus on women with at least one child is due to the fact that in India the transition from wife to mother occurs generally within two years after the marriage (Moore et al. 2009). Including married women without children may include women who cannot (themselves or their husbands) have a child and this could affect the women’s family size preference likewise.

In the three PSUs, there were 1040 households in total at the time of interview, with 690 households where married women and their mother-in-law were living together and sharing the same kitchen (34 % of the households were not eligible for interview because they did not meet the selection criteria of mother-in-law residing with daughter-in-law). Among them, about 25 % (171 households) were not eligible according to the selection criterion of the woman having at least one living child. This led to 519 eligible households for interview. Out of these 519 households, 59 were not interviewed because either the woman or her mother-in-law was not present at the time of interview (41 cases) or they refused to be interviewed (18 cases). From the remaining households (460), a total of 450 pairs of women and mother-in-law were interviewed in order to fulfil the target sample of the survey. The interview process stopped when the sample of 450 households was achieved.

Interviews were conducted at the respondents’ home in order to offer a familiar and comfortable environment to interviewees. Women and her mother-in-law were interviewed at the same time, but in different places in order to avoid reporting bias. A pair of investigator was sent in one household at a time where one interviewed the woman and another interviewed the mother-in-law. Since most women were of younger age and were often not allowed to go outside, they were interviewed inside the home. Mothers-in-law, on the other hand, were interviewed either in the courtyard or in the so-called *Baranda*, a connecting area between the main entrance of the house and the outside courtyard. This way privacy of each of the respondents could be maintained.

Each of the 450 woman–mother-in-law pairs was interviewed using semi-structured questionnaires. The questionnaires contained a mix of closed- and open-ended questions. All variables used in the analysis are derived from closed questions. Women’s questionnaire asked information on household characteristics, husband’s socioeconomic and demographic profiles, individual demographic characteristics as well as fertility-related information, e.g. fertility preferences, fertility history and interaction with mother-in-law on fertility issues. Information on women’s mother (i.e. age, education and the number of children ever born) was also collected during the interviews to these women. Mother-in-law’s questionnaire asked information on mother-in-law’s characteristics (e.g. age and education), fertility history (the number of children ever born) and fertility preference for the woman under study (desired number of grandchildren from the daughter-in-law). Mother-in-law was asked “How many children, boys and girls, do you want your daughter-in-law to have?”.

The interview data were coded and computerized in CSPro 4.0 software. The sample used for the following analysis is reduced to 440 woman–mother-in-law pairs since we excluded the cases where mother-in-law’s preference for grandchildren was not reported (*n* = 6) or reported as “up to God” (*n* = 4). Although this latter case would be interesting to investigate, the number of observations was too small to perform a meaningful statistical analysis.

### Dependent Variable

**Fig. 2:**
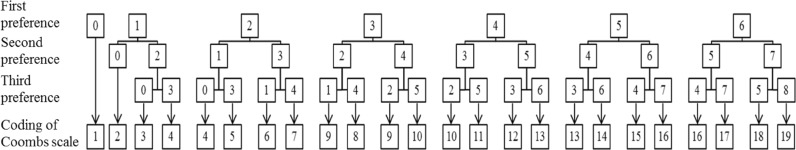
Response alternatives and coding scheme for Coombs scale of family size preference. Source: Authors’ adaptation from Axinn et al. (1994, p 70)

Desired family size of the woman is the dependent variable in this study, and it is classified into three categories: low; medium; and high. This variable is derived from the Coombs scale, a latent measure of desired family size considered to produce the most accurate indicator of fertility preferences (Coombs 1974, 1978, 1979). This scale is based on three follow-up survey questions. First, respondents were asked “If you could have the number of children you would like to have, what number of children would you want to have when your family size is completed?”. They were then asked two more questions, indicating whether the interviewed woman would prefer fewer or more children if she was unable to have the exact number previously indicated. For example, if the respondent answered three to the first question, she was then asked in the subsequent question whether she would prefer to have two (first answer minus one) or four (first answer plus one) children. In the final question, if she answered two in the second question, the respondent had to choose between one (second answer minus one) and four (the highest number asked so far), while if she answered four, she had to express her preference between two (the lowest number so far) and five (second answer plus one) (see Fig. 2). The Coombs scale produces variance in responses, pushing respondents who, following the same example, reported they desire three children, to report whether they would prefer a minimum of three children or a maximum of three children. This variance allows for high accuracy in estimating family size preference, which cannot be captured using just one question.

Responses were coded, in agreement to the previous literature (Axinn et al. 1994; Coombs 1974; Jennings et al. 2012), into a scale with values ranging from 1 to 19, with a higher value indicating a preference for more children. For example, a respondent who says she prefers three children in the first question, and three is the maximum number of children she would prefer, receives a code of 7, as compared to a code of 10 for a respondent who says that three is the minimum number of children she would prefer. Figure 2 illustrates the answers in three steps and the coding scheme.

In our sample, responses to the first question varied from 1 to 6. Accordingly, the responses to the following two questions could range from 0 to 7 and 0 to 8, respectively. This would correspond to Coombs scale’s coding of 1–19. However, in our sample, none of the women interviewed gave 0 as their “alternative” desired number of children. Hence, the actual codes of the Coombs scale in our study range from 4 to 19, with a higher value indicating a preference for more children. Based on the tertile distribution of the codes, we created three categories for the dependent variable women’s preferred family size: low; medium; and high. The low category corresponds to the codes 4–7, the medium category corresponds to 8–10, and the high category includes values 11–19.

As shown in Table 1, on average the women interviewed desire 3.2 children when first asked about the number of children they would want to have when their family size is completed. On the Coombs scale, women’s preferred family size equals to the average of 9.2, suggesting that three may not be the maximum number of children that they would prefer.

**Table 1:** Sample characteristics

	Percent/Mean (SD)	*N*
*Woman’s characteristics*		
Preferred family size [mean (SD)]	3.2 (1.1)	440
Coombs scale [mean (SD)]	9.3 (3.0)	440
Age (years)		
16–24	27.3	120
25–29	28.2	124
30–34	44.5	196
Education		
Uneducated	15.5	68
1–5 years of schooling	21.4	94
6–10 years of schooling	49.5	218
11+ years of schooling	13.6	60
Age at marriage [mean (SD)]	17.3 (2.3)	440
Sons ever born [mean (SD)]	1.7 (1.2)	440
Daughters ever born [mean (SD)]	1.6 (1.2)	440
Experienced child loss		
No	93.6	412
Yes	6.4	28
Village		
Bareja	28.4	125
Nautan	27.7	122
Sheetal pur	43.9	193
*Husband’s characteristics*		
Education		
Uneducated	9.1	40
1–5 years of schooling	9.8	43
6–10 years of schooling	49.6	218
11+ years of schooling	31.6	139
Activity status		
Not working	31.4	138
Working	68.6	302
*Household’s characteristics*		
Economic status		
Low	33.0	145
Middle	33.0	145
High	34.1	150
Caste		
SC/ST	17.0	75
OBC	34.3	151
Other caste	48.6	214
Religion		
Hindu	90.2	397
Muslims	9.8	43
Number of household members [mean (SD)]	8.6 (3.4)	440
Brothers-in-law in household		
No	12.7	56
Yes	87.3	384
Sisters-in-law in household		
No	12.7	56
Yes	87.3	384
*Mother*-*in*-*law’s characteristics*		
Children ever born [mean (SD)]	5.4 (1.6)	440
Preferred number of grandchildren from daughter-in-law [mean (SD)]	4.1 (1.0)	440
Age (years)		
41–49	20.0	88
50–54	44.1	194
55–60	35.9	158
Education		
Uneducated	81.1	357
1–5 years of schooling	4.8	21
6+ years of schooling	14.1	62
Interaction with daughter-in-law on childbearing issues		
Never	25.9	114
Sometimes	51.6	227
More often	22.5	99
*Mother’s characteristics*		
Children ever born [mean (SD)]	5.3 (1.6)	440
Education		
Uneducated	84.3	371
1–5 years of schooling	8.4	37
6+ years of schooling	7.3	32
Educational gap between woman and husband		
Years education husband − years education wife [mean (SD)]	2.2 (4.8)	440
Wife’s education = husband’s education − both literate	15.0	66
Wife’s education = husband’s education − both illiterate	2.5	11
Wife’s education > husband’s education	19.8	87
Wife’s education < husband’s education	62.7	276
Educational gap between woman and mother-in-law		
Years education woman − years education mother-in-law [mean (SD)]	5.5 (4.8)	440
Woman’s educated education ≤ mother-in-law’s education	23.6	104
Woman’s education > mother-in-law’s education	76.4	336
Total	100	440

### Explanatory Variables

We use four key explanatory variables in order to test our hypotheses (H1, H2 and H3) on the influence of biological mother’s and mother-in-law’s fertility patterns, and mother-in-law’s preference for grandchildren on women’s desired family size. In addition, women’s education and the educational gaps between the woman and mother-in-law and between the woman and her husband are included to test the fourth hypothesis (H4). The distribution of the explanatory variables is presented in Table 1.

1. *Mother’s number of children ever born* In our sample, mother’s number of children ever born is on average 5.3.
2. *Mother’s education* was classified into three categories: uneducated (84.3 %); 1–5 years of schooling (8.4 %); and 5 years of schooling and above (7.3 %).
3. *Mother*-*in*-*law’s number of children ever born* On average, the number of children ever born to mother-in-law in our sample is 5.4, similar to mother’s number of children ever born.
4. *Mother*-*in*-*law’s preferred number of grandchildren* On average, the interviewed mothers-in-law replied they want about 4 grandchildren from the woman under study.
5. *Woman’s education* Based on the number of years of schooling reported by the women interviewed, we constructed four education categories: uneducated (15.5 %); 1–5 years of schooling (21.4 %); 6–10 years of schooling (19.5 %); and 11 years of schooling and above (13.6 %).
6. *Education gap between the woman and her husband* On average, husband has 2.2 years of schooling more than his wife. Husband’s education was classified into four categories similar to that of the woman. As for couple’s relative educational attainments, we considered whether the woman’s education is higher (19.8 % of couples), lower (62.7 % of couples) or equal to her husband’s education based on the original continuous variables indicating the number of years the woman and her husband spent in education. In the case of equal number of years, we additionally distinguished between couples where both wife and husband were literate (15 %) and couples where they were both illiterate (2.5 %).
7. *Education gap between the woman and her mother*-*in*-*law* On average, women have 5.5 years of schooling more than mother-in-law. A dummy variable was created taking the value of 0 if the woman’s education (measured as years of education) was less or equal to mother-in-law’s education and taking the value of 1 if it was greater than mother-in-law’s education.

### Control Variables

Table 1 shows the distributions of a range of characteristics of the woman, her spouse, her mother and her mother-in-law controlled for in the statistical analysis. For women’s individual characteristic, we included the number of sons and daughters ever born (measured continuously). In a context like northern India where son preference is particularly strong, sex composition of children can influence preferred family size likewise. The dummy variable for experienced child loss is also included since the woman may adjust her fertility preference according to how many children she already has as well as to her child loss experience. We also controlled for the woman’s age at the time of the survey grouped into three categories based on the sample distribution^1^ (16–24, 25–29, and 30–34 years) and for woman’s age at marriage (continuous, ranging from 12 to 26). Husband’s characteristics included education (classified into four categories: uneducated; 1–5 years of schooling; 6–10 years of schooling; and 11 or more years of schooling) and current activity status (which equals 1 if the husband was working at the time of the interview; 0 otherwise). Mother’s and mother-in-law’s educational attainments were on average lower than the attainment of the younger generation (i.e. the woman and her husband), therefore we classified them into three categories: uneducated; 1–5 years of schooling; and more than 5 years of schooling. Mother-in-law’s age was grouped into three categories (41–49, 50–54 and 55–60 years). Unfortunately, the age of the mother is not available from the survey. The frequency of interaction between the woman and mother-in-law on childbearing was reported as never; sometimes; and often.

We also controlled for household characteristics: household economic status (low; medium; and high); caste^2^ [Scheduled Caste (SC); Other Backward Caste (OBC); and other caste] and religion (which equals 1 if being Hindu; 0 otherwise, with 9.8 % of the latter being Muslim). Principal component analysis (PCA) was used to construct a wealth index (*α* = 0.84) based on information on the type of housing,^3^ household durable assets, livestock, land ownership and size of agricultural land (Filmer and Pritchett 2001; Gwatkin et al. 2007). The index was then divided into three categories based on tertiles. We also control for the number of household members (ranging from 2 to 27) and two dummies indicating whether there are brothers- and sisters-in-law living in the household. All models also include three dummies indicating the village where the interview was carried out (Bareja—reference; Nautan; and Sheetal Pur).

In alternative model specifications, we controlled for the age difference between wife and husband. Similarly, in order to account for generational differences, we also controlled for the age difference between the interviewed woman and her mother-in-law. These additional controls did not improve our models, and we therefore decided not to include these variables in the models reported in the paper (results available on request).

## Methods

Commonly, previous studies using the Coombs scale as an outcome variable used ordinary least squares (OLS) approach to estimate family size preference (Axinn et al. 1994). However, the codes of the Coombs scale are not on a continuous scale and do not follow a normal distribution, a necessary condition for OLS regression. Therefore, we carried out ordered logistic regression analysis using the three categories (low; medium; and high preferred family size) that we created. The ordered logistic approach is indeed an appropriate method for an ordinal response variable like ours. The likelihood ratio test showed that the proportional odds assumption was not violated by our data, i.e. the coefficients that describe the relationship between any two pairs of the outcome groups are statistically the same. Thus, the use of ordered logistic estimation is justified. We also performed a robustness check using OLS models to estimate family size preference treating Coombs scale as a continuous variable following the previous literature (results are available upon request). The significance levels and directions of the independent variables are the same as using the ordered logit models, which we believe are more suitable for the reasons explained above.

The estimates are presented as odds ratios (ORs) with 95 % confidence intervals and corresponding significance level. OR >1 indicates higher odds of preference for a larger family size, while OR <1 indicates preference for a smaller family size. The percentage change in odds for each unit increase in the independent variable is calculated using the formula: 100 × (expβ − 1). The results can be interpreted as follows: an OR is the proportional odds ratio for a one unit increase in a particular independent variable on preference for a large versus the combined medium and small family size, given that the other variables in the model are held constant. Likewise, we can interpret the OR as the odds of the combined categories large and medium versus small family size preference, under the constraint that the coefficients from each individual analysis (high vs. medium and low; high and medium vs. low) are equal while leaving the intercepts free to vary. Therefore, ordered logit can be interpreted as a logistic regression with binary outcome.

## Results

### Descriptive Results

First, we examine the relationships between women’s preferred family size and the other relevant characteristics. Table 2 presents family size preference constructed as tertiles of the Coombs scale across relevant demographic and socioeconomic characteristics of the woman and her significant others. Chi-square test (or ANOVA in case of continuous variables) was employed to test the level of significance of the differences between categories.

**Table 2:** Woman’s family size preference across background characteristics (row percentages) and *p* value

	Woman’s family size preference			*p* value
	Small	Medium	Large	
*Woman’s characteristics*				
Age (years)				
16–24	42.5	45.8	11.7	0.001
25–29	38.7	33.1	28.2	
30–34	29.6	39.8	30.6	
Education				
Uneducated	17.6	42.6	39.7	0.003
1–5 years of schooling	30.9	44.7	24.5	
6–10 years of schooling	39.9	38.1	22.0	
11+ years of schooling	48.3	33.3	18.3	
Age at marriage [mean (SD)]	17.4 (2.7)	17.1 (2.0)	17.3 (2.3)	0.399
Sons ever born [mean (SD)]	1.6 (1.2)	1.6 (1.1)	1.9 (1.3)	0.168
Daughters ever born [mean (SD)]	1.6 (1.2)	1.6 (1.2)	1.6 (1.1)	0.965
Experienced child loss				
No	35.9	39.8	24.3	0.647
Yes	32.1	35.7	32.1	
Village				
Bareja	42.4	43.2	10.4	0.022
Nautan	31.2	36.9	32.0	
Sheetal Pur	34.2	38.9	26.9	
*Husband’s characteristics*				
Education				
Uneducated	25.0	32.5	42.5	0.130
1–5 years of schooling	34.9	21.6	32.6	
6–10 years of schooling	35.3	39.4	25.2	
11+ years of schooling	39.6	43.9	16.5	
Activity status				
Not working	32.6	43.5	23.9	0.502
Working	37.1	37.7	25.2	
*Household’s characteristics*				
Economic status				
Low	30.3	37.9	31.7	0.113
Middle	35.9	40.0	24.1	
High	40.7	40.7	18.7	
Caste				
SC/ST	36.0	36.0	28.0	0.243
OBC	35.8	31.8	32.5	
Other caste	35.5	46.3	18.2	
Religion				
Hindu	36.5	39.5	23.9	0.373
Muslims	27.9	39.5	32.6	
Number of household members [mean (SD)]	8.4 (3.4)	8.4 (3.3)	9.1 (3.6)	0.137
Brothers-in-law in household				
No	35.7	42.9	21.4	0.792
Yes	35.7	39.1	25.3	
Sisters-in-law in household				
No	30.4	48.2	21.4	0.364
Yes	36.5	38.3	25.3	
*Mother*-*in*-*law’s characteristics*				
Children ever born [mean (SD)]	4.9 (1.7)	5.5 (1.5)	5.8 (1.5)	0.000
Preferred number of grandchildren [mean (SD)]	3.9 (1.0)	4.1 (1.1)	4.2 (1.0)	0.045
Age (years)				
41–49	35.2	47.7	17.0	0.199
50–54	36.1	35.1	28.9	
>55	35.4	40.5	24.1	
Education				
Uneducated	35.9	38.1	26.1	0.575
1–5 years of schooling	28.6	52.4	19.0	
6+ years of schooling	37.1	43.5	19.4	
Interaction with daughter-in-law on childbearing issues				
Never	28.9	43.0	28.1	0.164
Sometimes	39.2	40.1	20.7	
More often	35.4	34.3	30.3	
*Mother’s characteristics*				
Children ever born [mean (SD)]	5.1 (1.5)	5.4 (1.6)	5.4 (1.7)	0.119
Education				
Uneducated	33.2	39.1	27.8	0.002
1–5 years of schooling	37.8	48.6	13.5	
6+ years of schooling	62.5	34.4	3.1	
Educational gap between woman and husband				
Wife’s education = husband’s education − both literate	43.9	36.4	19.7	0.037
Wife’s education = husband’s education − both illiterate	18.2	18.2	63.6	
Wife’s education > husband’s education	36.8	34.5	28.7	
Wife’s education < husband’s education	34.1	42.8	23.2	
Educational gap between woman and MIL^a^				
Woman’s education ≤ MIL’s education	20.2	46.2	33.7	0.001
Woman’s education > MIL’s education	40.5	37.5	22.0	
Total (*N* = 440)	35.7	39.6	24.8	

*p* value is obtained from Chi-square tests for categorical variables and ANOVA test for continuous variables

^a^MIL refers to mother-in-law

Unsurprisingly, age is positively correlated with family size preference, with older women expressing higher preference for larger family size. Education, both of the woman and of her husband, is negatively associated with preference for larger family size. The desire for a smaller family grows with increasing years of education: 48.3 % of women in the most highly educated group (i.e. with more than 10 years of schooling) preferred a small family as compared to only 17.6 % of women with no schooling. Interestingly, family size preference is also significantly associated with biological mother’s education. About two-thirds of women whose mother has more than 6 years of schooling would like to have small family size.

When considering the difference in educational attainment between the woman and her husband, it appears that if they are both illiterate, the preference is mainly for a large family size. For couples where both partners are literate, on the other hand, women’s preference is for a small family size. In particular, if the woman is more educated than the husband, she reports more often a preference for a small family size. Unlike the association found in the case of educational attainment of biological mothers, education of mother-in-law is not significantly associated with women’s family size preference. However, when considering mother-in-law’s education relative to that of the woman, we find that when the daughter-in-law is more educated than mother-in-law, she is more likely to express a desire for a small family size.

### Multivariate Analysis

In Tables 3 and 4, we report the results from a series of ordered logistic models run to test the four hypotheses raised above. Model I aims to understand the direct relationship between biological mothers’ total fertility and desired family size of women, taking into account only mother’s education and total fertility. In Model II, we further added mother-in-law’s characteristics (i.e. total fertility, age, education and frequency of interaction with daughter-in-law on childbearing issues). In Model III, husband’s characteristics (i.e. education and current activity status) and household’s characteristics (i.e. household economic status, caste, religion, number of household members, presence of brothers-in-law and presence of sisters-in-law in the household) were added. The final model (Model IV) additionally includes individual characteristics (i.e. age, education, age at marriage, number of sons ever born, number of daughters ever born, whether the woman has experienced child loss and the village where the woman lives) along with the variables included in Models I, II and III.

**Table 3:** Odds ratio (95 % confidence intervals in brackets) from ordered logistic regression models predicting woman’s family size preference

	Model I	Model II	Model III	Model IV
*Mother*				
Children ever born	1.048	1.043	1.048	1.005
	[0.940, 1.168]	[0.934, 1.164]	[0.936, 1.173]	[0.895, 1.128]
Education (*Ref: uneducated*)				
1–5 years	0.745	0.729	0.768	0.834
	[0.397, 1.400]	[0.380, 1.401]	[0.391, 1.507]	[0.422, 1.648]
6+ years	0.303**	0.325**	0.363*	0.436*
	[0.143, 0.641]	[0.151, 0.699]	[0.165, 0.800]	[0.194, 0.977]
*Mother*-*in*-*law*				
Children ever born		0.978	0.951	0.978
		[0.876, 1.093]	[0.850, 1.065]	[0.870, 1.099]
Preferred number of grandchildren		1.208*	1.224*	1.244*
		[1.021, 1.430]	[1.032, 1.452]	[1.043, 1.483]
Age (*Ref: 41*–*49* *years*)				
50–54 years		1.249	1.216	1.023
		[0.780, 2.000]	[0.754, 1.961]	[0.621, 1.687]
55–60 years		1.085	0.996	0.859
		[0.656, 1.794]	[0.591, 1.676]	[0.503, 1.469]
Education (*Ref: uneducated*)				
1–5 years of schooling		1.552	1.549	1.787
		[0.674, 3.570]	[0.661, 3.630]	[0.748, 4.267]
6+ years of schooling		1.126	1.185	1.362
		[0.645, 1.967]	[0.664, 2.113]	[0.753, 2.462]
Interaction with daughter-in-law (*Ref: Never*)				
Sometimes		0.737	0.706	0.718
		[0.479, 1.135]	[0.454, 1.098]	[0.459, 1.124]
More often		0.93	0.854	0.892
		[0.556, 1.554]	[0.504, 1.448]	[0.521, 1.528]
*Husband*				
Education (*Ref: uneducated*)				
1–5 years of schooling			0.618	0.666
			[0.262, 1.454]	[0.280, 1.584]
6–10 years of schooling			0.483*	0.491*
			[0.240, 0.973]	[0.242, 0.994]
11+ years of schooling			0.446*	0.476^†^
			[0.202, 0.983]	[0.213, 1.064]
Activity status (*Ref. not working*)				
Working			1.061	1.051
			[0.713, 1.578]	[0.702, 1.572]
*Household*				
Economic status (*Ref: Low*)				
Middle			0.682	0.583
			[0.296, 1.572]	[0.246, 1.382]
High			1.223	1.116
			[0.405, 3.687]	[0.360, 3.465]
Caste (*Ref: SC/ST*)				
OBC			1.137	1.088
			[0.648, 1.992]	[0.614, 1.929]
Other caste			0.944	0.949
			[0.545, 1.634]	[0.544, 1.658]
Religion (*Ref: Hindu*)				
Muslims			1.193	1.34
			[0.637, 2.233]	[0.694, 2.586]
Number of household members			1.055*	1.058*
			[1.000, 1.113]	[1.001, 1.119]
Brothers-in-law in household			1.266	1.268
			[0.708, 2.265]	[0.705, 2.283]
Sisters-in-law in household			0.919	0.938
			[0.524, 1.613]	[0.530, 1.661]
*Woman*				
Age (*Ref: 16*–*24* *years*)				
25–29 years				1.964*
				[1.169, 3.301]
30–34 years				2.314***
				[1.429, 3.747]
Education (*Ref: uneducated*)				
1–5 years				0.546^†^
				[0.287, 1.036]
6–10 years				0.423**
				[0.232, 0.772]
11+ years				0.352**
				[0.160, 0.774]
Age at marriage				0.956
				[0.880, 1.038]
Sons ever born	1.097	1.104	1.106	0.968
	[0.936, 1.284]	[0.941, 1.295]	[0.941, 1.300]	[0.811, 1.155]
Daughters ever born	1.006	1	1.007	0.866
	[0.859, 1.178]	[0.852, 1.173]	[0.855, 1.186]	[0.724, 1.037]
Experienced child loss	1.087	1.019	1.112	1.108
	[0.521, 2.268]	[0.480, 2.165]	[0.508, 2.433]	[0.496, 2.477]
Village (*Ref. Bareja*)				
Nautan	1.48	1.533	1.492	1.41
	[0.892, 2.455]	[0.904, 2.598]	[0.447, 4.977]	[0.413, 4.819]
Sheetal Pur	1.346	1.346	1.909	2.293^†^
	[0.872, 2.075]	[0.861, 2.104]	[0.822, 4.436]	[0.960, 5.478]
*N*	440	440	440	440
Log likelihood	−464.636	−460.115	−454.066	−442.079

*** *p* < 0.001; ** *p* < 0.01; * *p* < 0.05; ^†^ *p* < 0.10

**Table 4:** Odds ratio (95 % confidence intervals in brackets) from ordered logistic regression models predicting woman’s family size preference, controlling for educational gaps

	Model I	Model II
*Mother*		
Children ever born	1.070	1.052
	[0.951, 1.203]	[0.935, 1.184]
Education (*Ref: uneducated*)		
1–5 years of schooling	0.836	0.793
	[0.422, 1.656]	[0.402, 1.564]
6+ years of schooling	0.390*	0.420*
	[0.174, 0.872]	[0.189, 0.931]
*Mother*-*in*-*law*		
Children ever born	1.306***	1.325***
	[1.160, 1.471]	[1.176, 1.493]
Preferred number of grandchildren	1.219*	1.229*
	[1.022, 1.453]	[1.030, 1.467]
Age (*Ref: 41*–*49* *years*)		
50–54 years	0.928	0.915
	[0.562, 1.532]	[0.552, 1.517]
55–60 years	0.844	0.805
	[0.496, 1.436]	[0.476, 1.361]
Education (*Ref: uneducated*)		
1–5 years of schooling	1.544	
	[0.630, 3.786]	
6+ years of schooling	1.305	
	[0.728, 2.339]	
Interaction with daughter-in-law (*Ref: never*)		
Sometimes	0.691	0.707
	[0.441, 1.082]	[0.450, 1.111]
More often	0.973	0.971
	[0.566, 1.671]	[0.564, 1.673]
*Husband*		
Education (*Ref: uneducated*)		
1–5 years of schooling		0.647
		[0.271, 1.548]
6–10 years of schooling		0.470*
		[0.230, 0.960]
11+ years of schooling		0.465
		[0.208, 1.040]
Activity status (*Ref. not working*)		
Working	1.036	1.087
	[0.693, 1.550]	[0.725, 1.629]
*Household*		
Economic status (*Ref: low*)		
Middle	0.535	0.569
	[0.225, 1.272]	[0.240, 1.347]
High	0.919	1.063
	[0.295, 2.866]	[0.344, 3.285]
Caste (*Ref: SC/ST*)		
OBC	1.274	1.282
	[0.711, 2.282]	[0.714, 2.303]
Other caste	1.055	1.105
	[0.603, 1.846]	[0.631, 1.934]
Religion (*Ref: Hindu*)		
Muslims	1.250	1.078
	[0.646, 2.420]	[0.556, 2.091]
Number of household members	1.054	1.057*
	[0.998, 1.113]	[1.000, 1.117]
Brothers-in-law in household	1.412	1.452
	[0.776, 2.569]	[0.797, 2.646]
Sisters-in-law in household	0.879	0.919
	[0.493, 1.566]	[0.515, 1.640]
*Woman*		
Age (*Ref: 16*–*24* *years*)		
25–29 years	2.300**	2.171**
	[1.371, 3.856]	[1.295, 3.640]
30–34 years	2.549***	2.549***
	[1.570, 4.138]	[1.565, 4.153]
Age at marriage	0.94	0.942
	[0.865, 1.021]	[0.867, 1.024]
Sons ever born	0.975	0.919
	[0.820, 1.159]	[0.770, 1.097]
Daughters ever born	0.871	0.825*
	[0.732, 1.035]	[0.691, 0.986]
Experienced child loss	1.209	1.278
	[0.548, 2.665]	[0.581, 2.812]
Village (*Ref. Bareja*)		
Nautan	1.546	1.312
	[0.452, 5.283]	[0.383, 4.494]
Sheetal Pur	2.417*	2.289^†^
	[1.016, 5.752]	[0.959, 5.463]
Educational gap between woman and husband (*Ref: both literate*)		
Wife’s education = husband’s education − both illiterate	3.862	
	[0.937, 15.91]	
Wife’s education > husband’s education	1.071	
	[0.543, 2.114]	
Wife’s education < husband’s education	1.086	
	[0.623, 1.894]	
Educational gap between woman and MIL^a^ (*Ref: *Woman's education ≤ *MIL*)		
Woman’s education > MIL’s education		0.447***
		[0.280, 0.714]
*N*	440	440
Log likelihood	−434.954	−430.015

*** *p* < 0.001; ** *p* < 0.01; * *p* < 0.05; ^†^ *p* < 0.10

^a^MIL refers to mother-in-law

 Similar to the descriptive findings, Table 3 shows that the number of children ever born of the biological mother has no significant relationship with women’s family size preference across the four multivariate models. However, in the case of mother-in-law, although mother-in-law’s number of children ever born is not significantly associated with the woman’s preferred family size, her preferred number of grandchildren is. For one additional grandchild wanted by mother-in-law for the daughter-in-law, the odds of the latter wanting a larger family size increase by about 1.2 times. In additional analyses (not shown, available on request), we further distinguish the number of grandchildren that mother-in-law would like the daughter-in-law to have by gender. The results were similar to those reported here, but hinting to a stronger effect of the number of boys desired by the mother-in-law.

Educational attainment of the biological mother and of the woman, but not those of the mother-in-law and husband, has an inverse significant association with the woman’s family size preference. The higher the education of the biological mother, the lower the size of family that the woman would like to have is. This effect remains statistically significant even in Model IV where women’s education is controlled for. Likewise, the odds of wanting a larger family size are reduced by 58 and 65 % (Model IV) for women with 6–10 years and 11 and more years of schooling, respectively, compared with their counterparts who have no schooling. Age and other predictors such as frequency of interaction with mother-in-law, husband’s activity status and household characteristics do not appear to have a significant relationships with preferred family size.

The subsequent analysis in Table 4 examines how educational gaps between wife and husband and between daughter-in-law and mother-in-law mediate women’s family size preference. The significant effect of women’s education on family size preference observed earlier in Table 3 is not included here as it is accounted for in the dummy variable “Education woman—mother-in-law”. Similarly, we also excluded the variable education of husband in Model I. Extending from Model IV in Table 3, Model I in Table 4 assesses the effect of the educational gap between wife and husband on the woman’s family size preference. We found no significant relationship between husband and wife educational gap and family size preference. Model II includes a dummy variable indicating whether the woman has higher education than her mother-in-law. We find that when a daughter-in-law has higher education than her mother-in-law, she would prefer to have a smaller family size.

Interestingly, when controlling for educational differences between husband and wife and between daughter-in-law and mother-in-law in Models I and II, respectively, the number of children ever born to mother-in-law became statistically significant and positive. This shows that the educational difference variables may be a negative confounder. That is, the sensitivity to mother-in-law’s number of children ever born on women’s family size preference depends on educational differences between the woman and her mother-in-law as well as between the woman and her husband. In other words, this means that a subgroup of women is particularly sensitive to mother-in-law’s fertility, and it is likely to be the one with lower education than mother-in-law. The same explanation applies to the case of controlling for the educational gap between wife and husband in Model I.

## Discussion

Using original interview data collected from a sample of 440 woman–mother-in-law pairs in rural areas of north-eastern India, we analysed how much the women’s family size preference is influenced by intergenerational transmission of fertility behaviours. Based on ordered logistic regression analysis adjusting for relevant individual, family members’ and household socioeconomic characteristics, the main findings from this paper are discussed below in correspondence to the hypotheses explicated above.

First, biological mother’s fertility behaviour has no significant association with women’s preferred family size, but fertility of mother-in-law does. This finding rejects H1, which followed the literature on Western societies and predicted a relationship between biological mother’s number of children ever born and women’s family size preference, but supports H2, which assumed that in the rural Indian context fertility of mother-in-law has greater influence than biological mother’s fertility in shaping a young woman’s fertility preferences. Our finding differs from the few previous studies on intergenerational fertility transmission that considered both biological mother and mother-in-law. These extant studies commonly found that the correlation between biological mother’s number of children ever born and women’s number of children ever born was stronger than that between mother-in-law’s number of children ever born and women’s number of children ever born (Reher et al. 2008). Nevertheless, this previous literature was carried out in the Western context with different kinship systems and living arrangements from rural India. In our study setting, where women moved to cohabit with her husband’s family at young age after marriage, socialization within the in-law family appears to be more influential than within her natal family.

Second, as predicted by H3, the number of grandchildren that mother-in-law would like the daughter-in-law to have is positively associated with women’s preferred family size. It may be thought that the strong correlation observed is due to mother-in-law deliberately choosing a daughter-in-law who has a similar fertility preference as herself. In fact, not only did almost all these marriages occur at young ages, but they were also often arranged without the participation of young people themselves, as typically practised in Bihar (IIPS and Population Council 2010). In rural Bihar, the initiative for arranged marriages is commonly taken by the family of the bride, who usually brings a dowry to the husband’s family (Srinivasan and Lee 2004). Since such arranged marriages are likely to be endogamous in terms of caste, social class and ethnic group, one could expect a similarity between preference of the family of origin and that of the husband’s family. However, it is usually the bride’s family who initiates arranged marriages, and this reduces the likelihood that mothers-in-law deliberately choose daughters-in-law who share the same fertility preference. We thus argue that the association between the desired number of grandchildren of mother-in-law and women’s family size preference is rather a product of social pressure by mother-in-law.

In rural Bihar’s kinship practice, as explained above, there is little room for young women’s autonomy over their own life and childbearing decisions (Char et al. 2010; Das Gupta et al. 2003). Nevertheless, upon testing our last hypothesis (H4), we find that when the woman is more educated than her mother-in-law, the preferred family size becomes smaller. This effect is statistically significant net of women’s individual education effect. Although we do not have a direct measure of women’s empowerment such as participation in household decision-making or mobility, comparing the relative level of education between the woman and mother-in-law allows us to proxy the degree of autonomy that the woman may have within the family, relying on previous evidence that women with little education are more subject to the traditional pattern of subordination vis-à-vis husband and in-laws (Mason 1997). This suggests that when women have some schooling, and, in particular, when their educational attainment is greater than that of their mother-in-law, their relative power is enhanced. Therefore, they may more successfully negotiate for and execute their preferences related to childbearing decisions.

Although we expected to see a propensity for smaller family size preference when the woman has higher education than her husband (H4), our results do not support this hypothesis. While husbands can in general be influential in decision-making on contraceptive use and the timing and the number of children, it is in contexts where men control the family’s assets and act as the head of household that they typically have more say on reproductive decisions (Das et al. 2013; Karra et al. 1997). In the considered household setting, however, mothers-in-law may play a greater role on the reproductive behaviour of young women than their husbands, who are also under the pressure of the older generation (Barua and Kurz 2001). Indeed, in the Indian setting, there is evidence that mothers-in-law influences decisions made by couples regarding family planning adoption or modern contraceptive use as well as the number of children that the couple should have (Hall et al. 2008). This explanation is hinted, for example, by an earlier study on Uttar Pradesh showing that while only 15 % of the pregnant women interviewed deferred decision-making about health issues to their husbands, as much as 56 % did to their mothers-in-law (Prakash et al. 1994). It is possible that family size preferences of the woman and her husband are closer than those of the woman and her mother-in-law. As shown in a study of couples’ fertility behaviour in Bihar and Uttar Pradesh, husbands and wives tend to agree on their desired family size (Unisa et al. 2004). If this is the case, it may not matter much whether women have greater autonomy as measured by the gap between their education and their husband’s education.

The finding that biological mother’s education but not mother’s fertility is significantly associated with women’s family size preference is also worth discussing. We speculate that education may be a mediating factor between mother’s fertility behaviour and women’s fertility preferences since higher educated mothers are also more likely to have lower number of children than their less educated counterparts. Furthermore, through social learning, education of significant others may have spillover effects on individuals’ behaviours. Evidence has shown, for example, that the presence of literate family members is a favourable factor for contraceptive use, and hints to the positive externality of education in communication and diffusion of ideas and knowledge through interpersonal networks (McNay et al. 2003).

Overall, our paper provides two main contributions to the literature. First, we consider and compare the influence of mother and mother-in-law on fertility preferences of young married women in rural Bihar. No study so far, to our knowledge, has compared how fertility behaviours of both mother and mother-in-law can influence a woman’s preferred number of children in a developing country context. Moreover, our study setting is rather unique, and it allows us to test the dynamics of socialization both in natal family and in the in-law family. Second, we propose an innovative approach to study fertility preferences, measuring desired family size of women by Coombs scale and analysing it with an ordinal logistic model. For the first time to our knowledge, the ordinal categorical nature of the Coombs scale is addressed with a more appropriate estimation procedure.

We acknowledge that this study has some limitations mainly due to the nature of the survey design. First, as a result of the sample selection of the survey used, we can only consider women who have at least one child. We, however, believe that such selection does not lead to biased results because in the rural northern Indian context considered, childbearing is concentrated at age 20–29 (IIPS and ORC Macro 2000). In fact, at the turn of the century, only about 5 % of women aged 21–49 were childless in Bihar (Agrawal et al. 2012). Second, by interviewing only married women who reside with their mother-in-law, we are not able to compare their family size preferences with those of their counterparts who do not live with their husband’s family. Indeed, a recent study on contraceptive use in the urban area of Uttar Pradesh, India, demonstrated that women who live in the husband’s natal home with the mother-in-law were less likely to use family planning as compared to other types of living arrangements (Speizer et al. 2015). Our findings therefore are not generalizable to all India, but they are applicable to a large part of the rural population. Third, this study is based on a rather small sample size, and some effects may not achieve statistical significance because the sample size is too small. Fourth, the survey contains no information on family size preference of the husband, which other studies have shown to also be important (Mason and Smith 2000; Tilahun et al. 2014). Likewise, fertility preference of women’s mothers can also influence women’s family size preference, but this information is not available in the survey data used. In general, we acknowledge a lack of information about the woman’s mother that could help us in further understanding similarities and differences between the family of origin and the husband’s family. Our results thus are not entirely conclusive, and this suggests that further research into the issue is needed. In addition, a very small proportion of mothers-in-law (*n* = 4) reported “up to God” as their preference for the number of grandchildren. It would be interesting for future research with larger sample size to examine this subgroup of people and compare such response between different generations.

We also recognize that fertility preferences may not be the same as fertility behaviour. Since women interviewed in our survey are still in reproductive age, we focused on their fertility preferences controlling for the number of children ever born. Young women’s preferences of family size are, nevertheless, recognized to be one of the main determinants of achieved fertility (Barber 2001). Thus, looking at family size preferences rather than the actual number of children ever born should not be problematic in developing understanding of fertility behaviour.

## Conclusions

In such a unique social setting where a married woman lives and shares a kitchen with her mother-in-law like in our study, we found that both fertility and preference for the number of grandchildren of mother-in-law are associated with the preferred family size of women, but not the fertility of biological mother. This shows how sociocultural differences in family values and norms shape the patterns of intergenerational fertility transmission in a context where patriarchal joint family is commonly practised like in northern India.

Likewise, our findings show that several forces driving down the preference for large families operate beyond the individual circumstances. Our data show that the socioeconomic environment where women grew up, proxied by the level of education of their mother, has a persistent effect on women’s preferences. Moreover, the effect of both socialization with and social pressure from the husband’s family on women’s family size preferences is clear when looking at the positive effect of mother-in-law’s preferred number of grandchildren. On the other hand, even after accounting for these factors, the repressive effect of women’s education (and of their educational attainment relative to those of husband and mother-in-law) on their preferred family size remains significant, indicating that women’s education plays an important part. Improvements in the status and empowerment of women are central to the process of sustainable development. It is likely that when the level of women’s education increases, they will achieve autonomy to manage the resources available to them as well as play an active and effective role in family planning. Improved education and possibly job opportunities outside in-laws’ home may boost women’s status and consequently reduce the overall fertility rate in rural India.

## Footnotes

1 The age distribution is a result of the selection criteria of the sample of women sample currently living together with mother-in-law. Given that female life expectancy in India in 2010 was 65.7 (Goujon et al. 2012), including women aged >34 years in the study sample would encounter the problem of not having surviving mothers-in-law available for an interview.

2 Scheduled Caste (SC) includes “untouchables” or *Dalits*—a group that is socially segregated and economically disadvantaged; Other Backward Caste (OBC) is an intermediate caste, considered at a low level in the traditional caste society but above the SC; and “Other caste” (also called as upper caste or generals) is the remaining population, and it is characterized by better socioeconomic status than the SC and OBC populations. The Hindu society in India also includes the Scheduled Tribe (ST) that lives geographically isolated, with limited economic and social interactions with the rest of the population and therefore not present in the villages considered in this study. Our survey asked all respondents to identify their caste, independently of whether the respondent was Hindu or not.

3 Type of housing distinguishes between *Pucca* (roof, wall and floor are made of bricks, cement and rod), *Semi-pucca* (roof or wall or floor are made of bricks, cement and rod) and *Kaccha* (roof, wall and floor are not made of bricks, cement and rod). In the survey, this information is collected by observation of the interviewers.

## References

[CR1] Agrawal P, Agrawal S, Unisa S (2012). Spatial, socio-economic and demographic variation of childlessness in India: A special reference to reproductive health and marital breakdown. Global Journal of Medicine and Public Health.

[CR2] Ajzen I (1991). The theory of planned behaviour. Organizational Behavior and Human Decision Processes.

[CR3] Anderton DL, Tsuya NO, Bean LL, Mineau GP (1987). Intergenerational transmission of relative fertility and life course patterns. Demography.

[CR4] Asphjell, M. K., Hensvik, L., & Nilsson, J. P. (2013). *Businesses, buddies, and babies: Fertility and social interactions at work* (Working Paper Series, Center for Labor Studies No. 2013:8). Uppsala: Department of Economics, Uppsala University. https://ideas.repec.org/p/hhs/uulswp/2013_008.html. Accessed March 16, 2015.

[CR5] Axinn WG, Clarkberg ME, Thornton A (1994). Family influences on family size preferences. Demography.

[CR6] Balbo N, Barban N (2014). Does fertility behavior spread among friends?. American Sociological Review.

[CR7] Bankole A, Singh S (1998). Couples’ fertility and contraceptive decision-making in developing countries: Hearing the man’s voice. International Family Planning Perspectives.

[CR8] Barber JS (2001). The Intergenerational transmission of age at first birth among married and unmarried men and women. Social Science Research.

[CR9] Barber JS, Axinn WG (1998). The impact of parental pressure for grandchildren on young people’s entry into cohabitation and marriage. Population Studies.

[CR10] Barua A, Kurz K (2001). Reproductive health-seeking by married adolescent girls in Maharashtra, India. Reproductive Health Matters.

[CR11] Basu, A. M. (2002). Why does education lead to lower fertility? A critical review of some of the possibilities. *World Development*, *30*(10), 1779–1790. Accessed September 19, 2014

[CR12] Bengtson VL (1975). Generation and family effects in value socialization. American Sociological Review.

[CR13] Ben-Porath Y (1975). First-generation effects on second-generation fertility. Demography.

[CR14] Bernardi L (2003). Channels of social influence on reproduction. Population Research and Policy Review.

[CR15] Bhat PNM, Zavier AJF (2003). Fertility decline and gender bias in. Demography.

[CR16] Bhatnagar, I., Khan, M. E., & Hazra, A. (2013). Increasing postpartum contraception in rural Bihar. In M. Khan, F. Donnay, U. K. Tarigopula, & K. Aruldas (Eds.), *Shaping demand and practices to improve family health outcomes: Findings from a quantitative survey* (pp. 128–164). New Delhi: Population Council. http://iussp.org/en/event/17/programme/paper/3296. Accessed May 3, 2015.

[CR17] Bloom SS, Wypij D, Gupta MD (2001). Dimensions of women’s autonomy and the influence on maternal health care utilization in a north Indian city. Demography.

[CR18] Bongaarts J (2003). Completing the fertility transition in the developing world: The role of educational differences and fertility preferences. Population Studies.

[CR19] Bongaarts J (2010). The causes of educational differences in fertility in Sub-Saharan Africa. Vienna Yearbook of Population Research.

[CR20] Borkotoky K, Unisa S (2014). Female education and its association with changes in socio-demographic behaviour: Evidence from India. Journal of Biosocial Science.

[CR21] Bühler C, Philipov D (2005). Social capital related to fertility: Theoretical foundations and empirical evidence from Bulgaria. Vienna Yearbook of Population Research.

[CR22] Cain M, Khanam SR, Nahar S (1979). Class, patriarchy, and women’s work in Bangladesh. Population and Development Review.

[CR23] Chan CG, Elder GH (2000). Matrilineal advantage in grandchild–grandparent relations. The Gerontologist.

[CR24] Char A, Saavala M, Kulmala T (2010). Influence of mothers-in-law on young couples’ family planning decisions in rural India. Reproductive Health Matters.

[CR25] Cialdini, R. B., Reno, R. R., & Kallgren, C. A. (1990). A focus theory of normative conduct: Recycling the concept of norms to reduce littering in public places. *Journal of Personality and Social Psychology*, *58*(6). http://psycnet.apa.org/?&fa=main.doiLanding&doi=10.1037/0022-3514.58.6.1015.

[CR26] Ciliberto, F., Miller, A., Skyt Nielsen, H., & Simonsen, M. (2013). *Playing the fertility game at work: An equilibrium model of peer effects* (MPRA Paper No. 45914). Munich, Germany. http://mpra.ub.uni-muenchen.de/45914/. Accessed March 16, 2015. 10.1111/iere.12177 PMC5010872 27605729

[CR27] Coombs LC (1974). The measurement of family size preferences and subsequent fertility. Demography.

[CR28] Coombs LC (1978). How many children do couples really want?. Family Planning Perspectives.

[CR29] Coombs LC (1979). Underlying family-size preferences and reproductive behavior. Studies in Family Planning.

[CR30] Daniel EE, Masilamani R, Rahman M (2008). The effect of community-based reproductive health communication interventions on contraceptive use among young married couples in Bihar, India. International Family Planning Perspectives.

[CR31] Das N (1987). Sex preference and fertility behavior: A study of recent Indian data. Demography.

[CR32] Das KC, Das K, Roy TK, Tripathy PK (2013). Incongruence and differentials in reporting ideal family size by the couples in India. Journal of Family Welfare.

[CR33] Das Gupta M, Zhenghua J, Bohua L, Zhenming X, Chung W, Hwa-Ok B (2003). Why is Son preference so persistent in East and South Asia? A cross-country study of China, India and the Republic of Korea. Journal of Development Studies.

[CR34] Dodoo FN-A (1993). A couple analysis of micro-level supply/demand factors in fertility regulation. Population Research and Policy Review.

[CR35] Dubas JS (2001). How gender moderates the grandparent–grandchild relationship: A comparison of kin-keeper and kin-selector theories. Journal of Family Issues.

[CR36] Dyson T, Moore M (1983). On kinship structure, female autonomy, and demographic behavior in India. Population and Development Review.

[CR37] Easterlin RA, Tilly C (1978). The economics and sociology of fertility: A synthesis. Historical studies of changing fertility.

[CR38] Elder GH (1977). Family history and the life course. Journal of Family History.

[CR39] Elder GH (1994). Time, human agency, and social change: Perspectives on the life course. Social Psychology Quarterly.

[CR40] Fikree FF, Khan A, Kadir MM, Sajan F, Rahbar MH (2001). What influences contraceptive use among young women in urban squatter settlements of Karachi, Pakistan?. International Family Planning Perspectives.

[CR41] Filmer D, Pritchett LH (2001). Estimating wealth effects without expenditure data-or tears: An application to educational enrollments in states of India. Demography.

[CR42] Goujon, A., KC, S., Potančoková, M., Butz, W. P., & Lutz, W. (2012). *Asian demographic and human capital data sheet 2012*. Laxenburg, Austria: International Institute for Applied Systems Analysis (IIASA). http://www.iiasa.ac.at/web/home/research/researchPrograms/WorldPopulation/PublicationsMediaCoverage/ModelsData/AsiaDataSheet2012.pdf.

[CR53] Graaf, P. M. de, & Kalmijn, M. (2001). Trends in the intergenerational transmission of cultural and economic status. *Acta Sociologica*, *44*(1), 51–66. Accessed September 18, 2014

[CR43] Gwatkin DR, Rutstein S, Johnson K, Suliman E, Wagstaff A, Amouzou A (2007). Socio-economic differences in health, nutrition, and population within developing countries: An overview. Nigerian Journal of Clinical Practice.

[CR44] Hall MAK, Stephenson RB, Juvekar S (2008). Social and logistical barriers to the use of reversible contraception among women in a rural Indian village. Journal of Health, Population, and Nutrition.

[CR45] IIPS. (2010a). *District Level Household and Facility Survey (DLHS*-*3), 2007*–*08: India*. Mumbai: International Institute for Population Sciences (IIPS). Accessed June 3, 2015

[CR46] IIPS. (2010b). *District Level Household and Facility Survey (DLHS*-*3), 2007*–*08:Bihar*. Mumbai: International Institute for Population Sciences (IIPS). Accessed 3 June 2015

[CR69] IIPS, & MoHFW. (2010). *District Level Household and Facility Survey under Reproductive and Child Health Project (DLHS*-*3). District Fact Sheet, 2007*–*08*. Mumbai: International Institute for Population Sciences.

[CR47] IIPS, Population Council (2010). Youth in India: Situation and needs 2006–2007, Executive Summary.

[CR79] IIPS, & ORC Macro. (2000). *National Family Health Survey (NFHS*-*2), 1998*–*99: India* (No. Vol. I). Mumbai: International Institute for Population Sciences.

[CR48] Imaizumi Y, Nei M, Furusho T (1970). Variability and heritability of human fertility. Annals of Human Genetics.

[CR49] Isiugo-Abanihe UC (1994). Reproductive motivation and family-size preferences among Nigerian men. Studies in Family Planning.

[CR50] Jejeebhoy SJ, Sathar ZA (2001). Women’s autonomy in India and Pakistan: The influence of religion and region. Population and Development Review.

[CR51] Jennings JA, Sullivan AR, Hacker JD (2012). Intergenerational transmission of reproductive behavior during the demographic transition. The Journal of Interdisciplinary History.

[CR52] Kadir MM, Fikree FF, Khan A, Sajan F (2003). Do mothers-in-law matter? Family dynamics and fertility decision-making in urban squatter settlements of Karachi, Pakistan. Journal of Biosocial Science.

[CR54] Karra MV, Stark NN, Wolf J (1997). Male involvement in family planning: A case study spanning five generations of a South Indian family. Studies in Family Planning.

[CR55] Keim, S., Klärner, A., & Bernadi, L. (2009). *Who is relevant? Exploring fertility relevant social networks* (No. WP-2009-001). Rostock: Max Planck Institute for Demographic Research. http://www.demogr.mpg.de/en/projects_publications/publications_1904/mpidr_working_papers/who_is_relevant_exploring_fertility_relevant_social_networks_3213.htm. Accessed March 16, 2015.

[CR56] Kohler HP (2001). Fertility and social interaction: An economic perspective.

[CR57] Kohler HP, Behrman JR, Watkins SC (2001). The density of social networks and fertility decisions: Evidence from south Nyanza district, Kenya. Demography.

[CR58] Kolk M (2014). Multigenerational transmission of family size in contemporary Sweden. Population Studies.

[CR59] Kravdal Ø (2002). Education and fertility in sub-Saharan Africa: Individual and community effects. Demography.

[CR60] Kuziemko, I. (2006). Is having babies contagious? Estimating fertility peer effects between siblings. Unpublished manuscript, New Jersey. https://www0.gsb.columbia.edu/faculty/ikuziemko/papers/fertility_11_29_06.pdf.

[CR61] Lyngstad TH, Prskawetz A (2010). Do siblings’ fertility decisions influence each other?. Demography.

[CR62] Manski CF, Mayshar J (2003). Private incentives and social interactions: Fertility puzzles in Israel. Journal of the European Economic Association.

[CR63] Martin SL, Tsui AO, Maitra K, Marinshaw R (1999). Domestic violence in northern India. American Journal of Epidemiology.

[CR64] Mason, K. O. (1997). *How family position influences married women’s autonomy and power in five Asian countries* (East-West Center Working Papers No. 89). Honolulu, HI: East-West Center.

[CR65] Mason KO, Smith HL (2000). Husbands’ versus wives’ fertility goals and use of contraception: The influence of gender context in five Asian countries. Demography.

[CR66] Masood Kadir M, Fikree FF, Khan A, Sajan F (2003). Do mothers-in-law matter? Family dynamics and fertility decision-making in urban squatter settlements of Karachi, Pakistan. Journal of Biosocial Science.

[CR67] McNay K, Arokiasamy P, Cassen R (2003). Why are uneducated women in India using contraception? A multilevel analysis. Population Studies.

[CR68] Miller WB, Pasta DJ (1995). Behavioural intentions: Which ones predict fertility behaviour in married couples. Journal of Applied Social Psychology.

[CR70] MoHFW. (2013). *Health and family welfare statistics in India 2013*. New Delhi: Ministry of Health and Family Welfare (MoHFW). https://nrhm-mis.nic.in/PubFWStatistics%202013/Complete%20Book.pdf.

[CR71] Montgomery MR, Casterline JB (1996). Social learning, social influence, and new models of fertility. Population and Development Review.

[CR72] Moore A, Singh S, Ram U, Remz L, Audam S (2009). Adolescent marriage and childbearing in India: Current situation and recent trends.

[CR73] Murphy MJ (1999). Is the relationship between fertility of parents and children really weak?. Social Biology.

[CR74] Murphy MJ (2012). Intergenerational fertility correlations in contemporary developing counties. American Journal of Human Biology.

[CR75] Murphy MJ (2013). The intergenerational transmission of reproductive behaviour: Comparative perspectives. The History of the Family.

[CR76] Murphy MJ, Knudsen LB (2002). The intergenerational transmission of fertility in contemporary Denmark: The effects of number of siblings (full and half), birth order, and whether male or female. Population Studies.

[CR77] Murphy MJ, Wang D (2001). Family-level continuities in childbearing in low-fertility societies. European Journal of Population/Revue européenne de Démographie.

[CR78] Office of the Registrar General and Census Commissioner, India. (2011). *Census of India 2011: Provisional population totals*. Bihar: Office of the Director of Census Operations. http://censusindia.gov.in/2011-prov-results/data_files/bihar/Provisional%20Population%20Totals%202011-Bihar.pdf.

[CR80] Pamuk ER, Fuchs R, Lutz W (2011). Comparing relative effects of education and economic resources on infant mortality in developing countries. Population and Development Review.

[CR81] Philipov, D., & Bernardi, L. (2012). Concepts and operationalisation of reproductive decisions implementation in Austria, Germany and Switzerland. *Comparative Population Studies*-*Zeitschrift für Bevölkerungswissenschaft*, *36*(2). http://www.comparativepopulationstudies.de/index.php/CPoS/article/view/78.

[CR82] Pink S, Leopold T, Engelhardt H (2014). Fertility and social interaction at the workplace: Does childbearing spread among colleagues?. Advances in Life Course Research.

[CR83] Pollet TV, Nelissen M, Nettle D (2009). Lineage based differences in grandparental investment: Evidence from a large British cohort study. Journal of Biosocial Science.

[CR84] Prakash A, Swain S, Negi KS (1994). Who decides?. Indian Pediatrics.

[CR85] Preston SH (1976). Family sizes of children and family sizes of women. Demography.

[CR86] Reher DS, Ortega JA, Sanz-Gimeno A (2008). Intergenerational transmission of reproductive traits in Spain during the demographic transition. Human Nature.

[CR87] RGI. (2011). *Maternal and child mortality and total fertility rates: Sample Registration System*. New Delhi: Office of the Registrar General & Census Commissioner, India (RGI).

[CR88] Säävälä M (2001). Fertility and familial power relations: Procreation in South India.

[CR89] Santhya, K. G., & Jejeebhoy, S. J. (2012). *The sexual and reproductive health and rights of young people in India: A review of the situation*. New Delhi: Population Council. http://www.popcouncil.org/uploads/pdfs/2012PGY_IndiaYouthSRHandRights.pdf. Accessed June 3, 2015.

[CR90] Schoen R, Astone NM, Kim YJ, Nathanson CA, Fields JM, Fields M (1999). Do fertility intentions affect fertility behavior?. Journal of Marriage and Family.

[CR91] Self S, Grabowski R (2013). Female autonomy in rural North India: Impact of economic, social and political factors. Journal of Economic Development.

[CR92] Singh A, Ram F, Ranjan R (2006). Couples’ reproductive goals in India and their policy relevance. Social Change.

[CR93] Speizer IS, Lance P, Verma R, Benson A (2015). Descriptive study of the role of household type and household composition on women’s reproductive health outcomes in urban Uttar Pradesh, India. Reproductive Health.

[CR94] Srinivasan P, Lee GR (2004). The dowry system in Northern India: Women’s attitudes and social change. Journal of Marriage and Family.

[CR95] Stephenson R, Hennink M (2004). Barriers to family planning service use among the urban poor in Pakistan. Asia-Pacific Population Journal.

[CR96] Thomson E (1997). Couple childbearing desires, intentions, and births. Demography.

[CR97] Tilahun T, Coene G, Temmerman M, Degomme O (2014). Spousal discordance on fertility preference and its effect on contraceptive practice among married couples in Jimma zone, Ethiopia. Reproductive Health.

[CR98] Unisa, S., Shekhar, S., & Roy, T. K. (2004). Husband-wife agreement on fertility goals: A study of three selected districts from Bihar and Uttar Pradesh. In T. K. Roy, M. Guruswamy, & P. Arokiasamy (Eds.), *Population Health and Development in India: Changing Perspectives* (pp. 157–176). New Delhi: Rawat Publications. http://www.abebooks.com/Population-Health-Development-India-Changing-Perspectives/409113924/bd. Accessed October 13, 2015.

[CR99] Upadhyay UD, Gipson JD, Withers M, Lewis S, Ciaraldi EJ, Fraser A (2014). Women’s empowerment and fertility: A review of the literature. Social Science and Medicine.

[CR100] Wilson-Williams L, Stephenson R, Juvekar S, Andes K (2008). Domestic violence and contraceptive use in a rural Indian village. Violence Against Women.

